# C-Reactive protein level and left ventricular mass are associated with acute cellular rejection after heart transplant

**DOI:** 10.6061/clinics/2021/e3020

**Published:** 2021-11-23

**Authors:** Débora Cestari Bacal, Miguel Morita Fernandes-Silva, Sandrigo Mangini, Marcia Santos de Jesus, Fernando Bacal

**Affiliations:** I Programa de Transplante Cardiaco, Hospital Israelita Albert Einstein (HIAE), Sao Paulo, SP, BR.; II Universidade Federal do Parana, Curitiba, PR, BR.

**Keywords:** Biomarkers, Heart Transplant, Inflammation, Ventricular Remodeling

## Abstract

**OBJECTIVES:**

Acute cellular rejection (ACR) remains a major complication of heart transplant (HT). The gold standard for its diagnosis is endomyocardial biopsy (EMB), whereas the role of non-invasive biomarkers for detecting ACR is unclear. This study aimed to identify non-invasive biomarkers for the diagnosis of ACR in patients undergoing HT and presenting with normal left ventricular function.

**METHODS:**

We evaluated patients who underwent HT at a single center between January 2010 and June 2019. Patients were enrolled after HT, and those with left ventricular (LV) systolic dysfunction before EMB were excluded. We included only the results of the first EMB performed at least 30 days after HT (median, 90 days). Troponin, B-type natriuretic peptide (BNP), and C-reactive protein (CRP) levels were measured and echocardiography was performed up to 7 days before EMB. ACR was defined as International Society for Heart and Lung Transplantation grade 2R or 3R on EMB. We performed logistic regression analysis to identify the non-invasive predictors of ACR (2R or 3R) and evaluated the accuracy of each using area under the receiver operator characteristic curve analysis.

**RESULTS:**

We analyzed 72 patients after HT (51.31±13.63 years; 25 [34.7%] women); of them, 9 (12.5%) developed ACR. Based on multivariate logistic regression analysis, we performed forward stepwise selection (entry criteria, *p*<0.05). The only independent predictors that remained in the model were CRP level and LV mass index. The optimal cut-off point for CRP level was ≥15.9 mg/L (odds ratio [OR], 11.7; *p*=0.007) and that for LV mass index was ≥111 g/m^2^ (OR, 13.6; *p*=0.003). The area under the receiver operating characteristic curve derived from this model was 0.87 (95% confidence interval [CI], 0.75-0.99), with sensitivity of 85.7% (95% CI, 42.1%-99.6%), specificity of 78.4% (95% CI, 64.7%-88.7%), positive predictive value of 35.3% (95% CI, 14.3%-61.7%), and negative predictive value of 97.6% (95% CI, 87.1%-99.9%).

**CONCLUSIONS:**

Among patients undergoing HT, CRP level and LV mass were directly associated with ACR, but troponin and BNP levels were not.

## INTRODUCTION

Heart transplant (HT) is the treatment of choice for patients with advanced heart failure (HF), who are refractory to optimal guideline-directed medical therapy because it significantly improves their survival outcome and quality of life (1). However, its use is limited by the low availability of organs and the resulting medical complications, such as primary graft dysfunction, right ventricular (RV) dysfunction, and acute rejection and infection (1-4).

Acute cellular rejection (ACR), which occurs in more than 30% of patients in the first year following HT, is characterized by an autoimmune response mediated by T cells with lymphocyte and macrophage infiltration, resulting in cardiomyocyte inflammation and necrosis (2,5,6). Because ACR can be asymptomatic, its early detection requires active monitoring with serial endomyocardial biopsy (EMB), the gold standard for its diagnosis (7,8). However, this invasive approach poses a challenge because of its high cost and risk of complications such as RV perforation, cardiac tamponade, arrhythmia, pneumothorax, and tricuspid valve injury (3,9-11). The currently accepted rejection classification after HT was proposed by the International Society of Heart and Lung Transplant (ISHLT) (12,13).

Non-invasive approaches have been studied that involve monitoring patients after HT without the risks associated with frequent EMB. Serum levels of biomarkers such as troponin and brain natriuretic peptide (BNP) can help identify myocardial injury and increased myocardial wall stress, respectively, characteristic pathological phenomena that occur in patients with ACR (14-19). Imaging methods such as echocardiography, myocardial scintigraphy, and cardiac magnetic resonance (1,20) yield information about changes in cardiac structure and function that may be associated with ACR (4,21-23). However, the role of these non-invasive methods in monitoring and detecting ACR after HT is uncertain since studies to date have been limited by small sample size, possible biases of determination, and collection of blood samples that did not follow the institutional protocol.

Therefore, we aimed to investigate whether clinical characteristics, serum biomarkers, and measures of cardiac structure and function can predict ACR in patients with normal LV ejection fraction following HT since the development of new ventricular dysfunction in the first few months after HT is related to humoral or cellular rejection.

## METHODS

This retrospective cohort study involved the medical record review of patients who underwent HT and subsequent EMB at the Heart Transplant Unit of Hospital Israelita Albert Einstein (HIAE) between January 2010 and January 2019. The inclusion criteria were having undergone HT at HIAE during the study period and the availability of complete ACR evaluation data. The exclusion criteria were death in the immediate postoperative period, ventricular dysfunction on echocardiogram, missing biomarker or echocardiogram covariate data, active infection or humoral rejection episodes, and persistent ACR.

**Figure 1:**
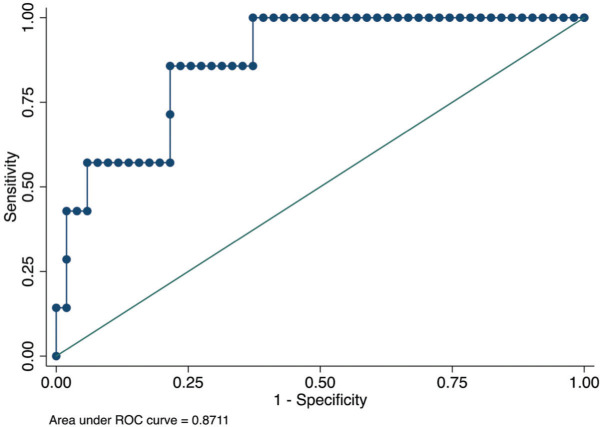
Receiver operating characteristic (ROC) curve of the ability of the multivariate model to predict the occurrence of acute cellular rejection.

From January 2010 to January 2019, all patients who underwent EMB were evaluated the same week that blood samples were drawn. According to the institutional protocol, every patient underwent EMB at 15 days, 30 days, and monthly thereafter for up to 6 months after HT (1). For this analysis, we evaluated only one EMB session per patient, which should be performed at least 30 days following HT for the detection of allograft ischemic injury and changes in the hemodynamic status or inflammatory response in the early phases after cardiac surgery. All EMB procedures were studied and defined according to the ISHLT 2005 classification (Figure 1).

The following data were collected:

– Clinical covariates: sex, age, etiology of cardiomyopathy, and death rate.
– Serum biomarkers: BNP, troponin I, and C-reactive protein (CRP) within 7 days of each EMB. Biomarkers were obtained before EMB to determine that the levels were not influenced by the procedure.
– Echocardiography performed immediately after EMB: left ventricular (LV) diastolic diameter, LV ejection fraction, LV septal and posterior wall thicknesses, RV function, pericardial effusion, cardiac mass, and cardiac mass index.
– EMB.

Biomarkers were collected according to these methods:

– Troponin I by chemiluminescence immunoassay method (normal, <34 pg/mL).
– BNP by chemiluminescence immunoassay (normal, 5-100 pg/mL).
– CRP by the turbidimetric method (reference value, <5 mg/L).

Serum biomarker determination and echocardiography were performed according to the biopsy routine. The biopsies were submitted for direct visualization and immunohistochemical analysis to exclude reactivation of Chagas disease. Biopsies were only performed at this early phase when an adequate serum level of a calcineurin inhibitor (cyclosporine>300 pg/mL or tacrolimus>10 pg/mL) was confirmed.

### Ethical consideration

The study was approved by the Ethics Committee of HIAE (number 16847519.2.0000.0071) and registered with Sistema Gerenciador de Projeto de Pesquisa (number 3681-19).

### Statistical analysis

Continuous data were evaluated for normal distribution and are presented as mean±standard deviation or median (25^th^-75^th^ percentile). Comparisons of clinical characteristics, serum biomarkers, and echocardiography data between patients with and without ACR was performed using Student's t-test, Mann-Whitney U test, or chi-squared test, as appropriate.

We then performed univariate logistic regression analysis of the predictors significantly associated with ACR and generated a receiver operating characteristic (ROC) curve to evaluate the discriminatory power and identify the cut-off point with optimal sensitivity and specificity. Finally, we performed logistic regression analysis with the significant predictors as dichotomous covariates using the respective cut-off points. The level of significance was set at *p*-value of <0.05.

Next, we selected the covariates with *p*-values of <0.10 from the univariate analysis and included them in automated stepwise forward selection to identify the independent predictors of acute rejection. We then generated an ROC curve and selected the optimal cut-off point based on the maximum Youden’s index and calculated the respective sensitivity, specificity, positive predictive value (PPV), and negative predictive value (NPV).

## RESULTS

From January 2010 to January 2019, a total of 107 patients underwent HT. We excluded 35 patients: five died within 30 days after HT, 29 were lacking laboratory results, and one missed an EMB. Therefore, we ultimately included 72 patients, among whom 9 (12.5%) developed ACR. The median follow-up period after HT was 90 (interquartile range, 51-158.5) days.

Compared with patients without ACR, those with ACR displayed higher blood CRP levels (7.1 *versus* 22 mg/L; *p*=0.046), whereas echocardiography measures indicated an increased LV mass (septal [10.75 *versus* 12.22 mm; *p*=0.021], posterior wall thickness [10.41 *versus* 11.56 mm; *p*=0.033], and LV mass index [94.72 *versus* 117.81 g/m^2^; *p*=0.004]). All other characteristics were similar between groups, including biomarkers, which did not differ significantly. However, we must consider that the BNP levels were missing for 10 patients, troponin levels were missing for 18 patients, and CRP levels were missing for 14 patients (Table 1).

We also found no association between pericardial effusion (PE) and ACR. Echocardiography was performed on the day that EMB was performed to screen for possible complications such as PE that can occur after the procedure.

A more detailed analysis of the significant variables in the study was performed using logistic regression analysis. The association of each predictor with the presence of ACR according to the cut-off point is shown in Table 2. These results demonstrate that, although the variables are significant, the confidence interval for each is not narrow. In addition, the analysis of the LV mass index exerts the greatest power for detecting post-HT ACR (OR=13.46).

For the multivariate logistic regression analysis, we performed a forward stepwise selection (entry criteria *p*<0.05) including the etiology of HF, CRP level, LV mass index, and LV septal and posterior wall thicknesses. The only independent predictors that remained in the model were CRP level (odds ratio [OR], 1.06; 95% confidence interval [CI], 1.01-1.12, *p*=0.025) and LV mass index (OR, 1.05; 95% CI, 1.01-1.10; *p*=0.021). This model derived the following equation to predict the log odds of ACR:

Rejection log odds=−8.656201+CRP (mg/L)×0.0622941+LV mass index (g/m^2^)×0.051881.

The area under the ROC curve derived from this model was 0.87 (95% CI, 0.75-0.99) (Figure 1). The *p*-value (Hosmer and Lemeshow test) was 0.90, although the sample size was too small to evaluate the model calibration.

The rejection log odds of −2.17 was the optimal cut-off point based on the maximum Youden’s index value. Using this cut-off point, we found sensitivity of 85.7% (95% CI, 42.1%-99.6%), specificity of 78.4% (95% CI, 64.7%-88.7)%, PPV of 35.3 (95% CI, 14.3%-61.7%), and NPV of 97.6% (95% CI, 87.1%-99.9%).

From the clinical point of view, a patient with a rejection log odds of ≥−2.17 has a CRP level of ≥15.9 mg/L and/or an LV mass index of ≥111 g/m^2^. The presence of both biomarkers above these cut-off points increased the predicted probabilities of ACR (Table 3).

**Figure 2:**
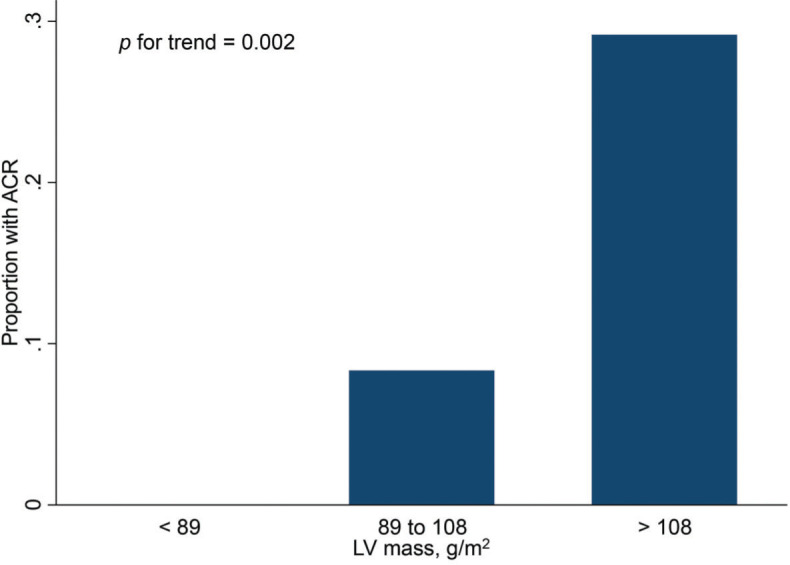
Proportion of ACR cases by LV mass tertile. ACR, acute cellular rejection; LV, left ventricular.

**Figure 3:**
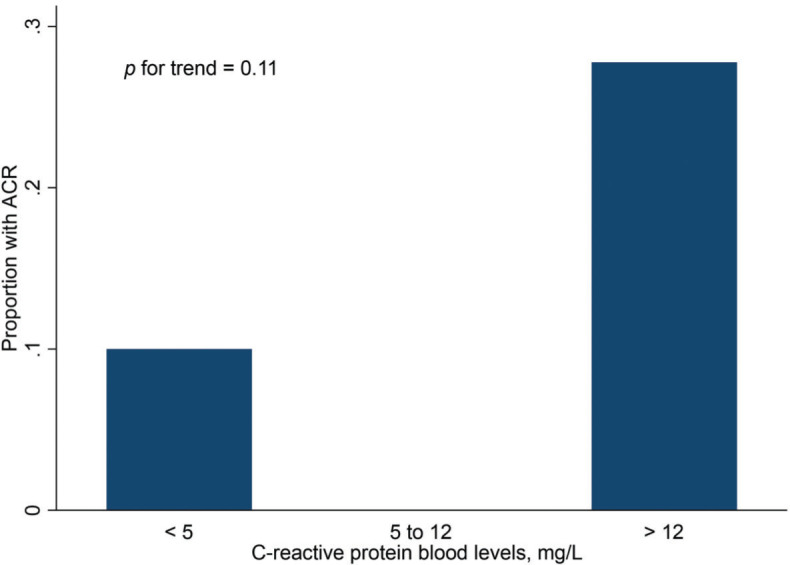
Proportion of ACR cases by C-reactive protein level tertile. ACR, acute cellular rejection.

Finally, Figures 2 and 3 show the distribution of ACR episodes and their relationship with LV mass and CRP, respectively. These data reinforce the importance of echocardiographic changes, particularly remodeling data, signaling ACR episodes, and the inflammatory biomarker CRP, which can also reflect the inflammatory process that occurs in rejection, excluding active infection at the time of evaluation.

## DISCUSSION

As the main findings of our study, we observed that after 30 days of postoperative HT, CRP, LV indexed mass, septal thickness, and LV posterior wall thickness non-invasively predicted ACR in patients with a preserved ventricular function and no clinical suspicion of rejection. In addition, we found a low PPV but a very high NPV. We can conclude that a CPR level of <15.9 mg/L and an LV mass index of <111 g/m^2^ can eliminate the need for EMB in cases of low clinical suspicion. This main finding has not been described in this way in the literature until now.

When analyzing the rejection episodes and their respective etiologies, we found a greater tendency toward ACR in the Chagas population after 30 days. It is important to mention that because of the use of immunosuppressors, the reactivation of Chagas disease can occur in the new graft, which can be characterized as rejection. However, direct visualization and immunohistochemical analysis in EMB were performed for differentiation if the infiltrate level is higher than 2R since the treatments differ (1,5,24-27).

In contrast to our study, other authors reported that levels of biomarkers such as BNP and troponin could play a role in ruling out ACR in patients after HT (19,20). Moreover, in patients who undergo HT, elevation in BNP levels despite normal ventricular function has been reported. Previous studies demonstrated that both LV and RV free wall longitudinal strain may exclude ACR and reduce the burden of repeated EMB (9). Finally, tissue Doppler velocity, an echo parameter, can rule out ACR (23).

Surgery usually causes an inflammatory state that persists in the first weeks of follow-up along with the inflammation resulting from cardiopulmonary bypass, causing high CRP levels during this period (5). Relative BNP changes after each EMB were associated with the presence of cell rejection in other series since BNP and pro-BNP levels are elevated in patients who undergo HT compared with those in normal individuals. Intra-individual changes in BNP level over time, which reflect the difference between BNP measurements taken at the time of two consecutive biopsies rather than absolute values, were considered more effective at detecting graft dysfunction. This is because of interindividual variety in BNP levels, a factor that makes it difficult to identify a single useful threshold for the diagnosis of rejection (28,29). The increase in BNP levels during acute graft rejection can be partially explained by the ability of pro-inflammatory cytokines such as tumor necrosis factor-α and interleukin-1 to increase BNP-promoting activity, modulate BNP gene expression in cardiomyocytes, and increase BNP secretion by activating protein kinase signaling (9,13).

However, despite being synthesized by the myocardium, serum BNP levels can be altered by non-cardiac factors, such as renal function, immune system disorders, and hypoxic conditions in addition to neuroendocrine factors that can modulate its secretion, including adrenergic agonists, glucocorticoids, endothelin-1, acetylcholine, prostaglandins, thyroid hormones, and angiotensin II. This explains why BNP levels may remain elevated after HT, even in the absence of a hemodynamic disorder of the LV or allographic rejection (9,11-13,19-21,30-32).

Level of troponin, the other biomarker studied here, was an important limitation of HT rejection screening. This can be explained by the lack of an association between biopsy-proven rejection and cardiac troponin T and cardiac troponin I levels in the first 2 months after HT since these levels may remain elevated in all cases because of factors such as ischemia of the donor heart and perioperative myocardial injury (11,12,18,20,21,33).

Studies of non-invasive methods aim to reduce the number of EMB procedures required and correlate biomarkers with ACR scenarios. Consequently, they intend to reduce health care costs and the inherent risks of procedures that can decrease the survival rate of HT patients (3,19).

The echocardiographic changes that we found, particularly the variations in ventricular thickness and mass, can also be useful in the follow-up of HT patients. These findings are representative of myocardial edema, which often occurs in episodes of ACR because of the inflammatory process, a result of immune system assault of the graft. The possibility of obtaining this comparative and sequential information can aid in the identification of patients who should be referred for EMB to confirm rejection and early institution of specific treatment (12,13).

We also found a correlation between CRP level and ACR episodes in the late follow-up period after HT. In the most immediate period, because of the postoperative inflammatory process, this variable has less discriminatory power for signaling ACR episodes. It is important to emphasize that CRP levels can be elevated in infectious processes with viral, bacterial, or fungal etiologies. Patients who underwent a complete analysis including biopsy, biomarker determination, and echocardiographic parameter evaluations did not present any signs of infection, which was an exclusion criterion in previous studies (4,34).

Our study has some limitations. First, it was a unicenter study with a small number of patients undergoing HT, which led to a small rate of ACR episodes. Second, it is possible that healthier patients were selected for the study since those with ventricular dysfunction were excluded, which could have introduced selection bias. To confirm our findings, this strategy must be externally validated in large prospective studies.

## CONCLUSION

Based on our study objectives and results, we identified non-invasive methods that indicated the presence of ACR after the first month of follow-up. Among patients undergoing HT, CRP level and LV mass were associated with ACR. The real application of these non-invasive methods to predict ACR, which has the potential to avoid unnecessary biopsies, must be confirmed in other prospective studies with a greater number of patients and ACR episodes.

## AUTHOR CONTRIBUTIONS

Bacal DC participated in the research design, data collection, data analysis, and manuscript writing processes. Fernandes-Silva MM participated in the research design, research performance, data analysis, and manuscript writing processes. Mangini S participated in the data analysis and manuscript writing processes. Jesus MS participated in the data collection. Bacal F participated in the research design, research performance, data analysis, and manuscript writing processes.

## Figures and Tables

**Table 1:** Serum biomarkers and echocardiography parameters for ACR detection.

Variables	First biopsies after 30 days		
	No rejection (n=63)	Rejection (n=9)	*p*-value
Age (years)	52.03±13.0	46.22±17.47	0.23
Etiology		0.07	
Ischemic	18 (28.6%)	1 (11.1%)	
Chagasic	12 (19.0%)	5 (55.6%)	
Dilated	20 (31.7%)	3 (33.3%)	
Other	13 (20.6%)	0 (0%)	
Female sex (n)	22 (34.9%)	3 (33.3%)	0.93
BNP (pg/mL)	138.0 [66.0, 407.0]	289.0 [152.0, 423.0]	0.13
Troponin (ng/mL)	0.0 [0.0, 0.1]	0.1 [0.0, 0.1]	0.31
CRP (mg/L)	7.1 [2.5, 12.6]	22.0 [2.5, 40.1]	**0.046**
DD (mm)	45.00±5.15	46.22±3.99	0.50
LVEF (%)	65.87±8.23	60.22±6.57	0.05
LV mass (g)	167.08±38.26	203.54±21.86	**0.007**
LV mass index (g/m^2^)	94.72±22.87	117.81±12.21	**0.004**
Septal thickness (mm)	10.75±1.79	12.22±1.48	**0.021**
PW thickness (mm)	10.41±1.53	11.56±0.88	**0.033**
RVDysf (n)	9 (14.3%)	2 (22.2%)	0.54
PE (n)		0.35	
Absent	49 (77.8%)	5 (55.6%)	
Mild	10 (15.9%)	3 (33.3%)	
Moderate	4 (6.3%)	1 (11.1%)	
Severe	0 (0%)	0 (0%)	

BNP, B-type natriuretic peptide; CRP, C-reactive protein; DD, diastolic diameter; LVEF, left ventricular ejection fraction; LV mass, left ventricular mass; LV mass index, left ventricular mass index; PE, pericardial effusion; PW, posterior wall; RVDysf, right ventricular dysfunction.

**Table 2:** Predictors of rejection after 30 days on univariate regression analysis.

Variable	Odds ratio	95% CI	Coefficient	Standard error	*p*-value
CRP≥15.9 mg/L	11.66	1.94-69.93	2.69	10.66	0.007
LV mass≥192 g	9.47	1.78-50.16	2.64	8.05	0.008
LV mass index≥111 g/m^2^	13.46	2.49-72.64	3.02	11.57	0.003
Septal thickness≥12 mm	8.10	1.53-42.67	2.47	6.86	0.014
PW thickness ≥11 mm	8.8	1.03-74.55	1.99	9.59	0.046

Odds ratios were adjusted for CRP, LV mass, septal thickness, and PP thickness.

CI, confidence interval; CRP, C-reactive protein; LV mass, left ventricular mass; LV mass index, left ventricular mass index; PW, posterior wall.

**Table 3:** Predicted probabilities of ACR by CRP level and LV mass index.

Parameter	Probability of ACR	95% CI
↓ CRP and ↓ LV mass index	0%	0%-11%
↑ CRP or ↑ LV mass index*	14%	4%-36%
↑ CRP and ↑ LV mass index	80%	28%-99%

* Only one biomarker was above the cut-off value.

ACR, acute cellular rejection; ↑ CRP, C-reactive protein≥15.9 mg/L; ↑ LV mass index, left ventricular mass index≥111 g/m^2^.
